# The Impact of COVID-19 on Burnout, Psychological Well-being, and Work Satisfaction in Psychiatry Trainees in Ireland

**DOI:** 10.1007/s40596-022-01633-0

**Published:** 2022-04-19

**Authors:** Caoimhe Mcloughlin, Ahad Abdalla, Aoife K. O’Callaghan, Sarah Casey, Elizabeth Barrett

**Affiliations:** 1 University of Edinburgh/Royal Infirmary Edinburgh, Edinburgh, Scotland; 2 University of Edinburgh, Edinburgh, Scotland; 3 Tallaght University Hospital, Dublin 24, Ireland; 4 Daughters of Charity Disability Services, Dublin 7, Ireland; 5 University College Dublin/Children’s University Hospital, Dublin 1, Ireland

**Keywords:** Burnout, Education and training, Well-being, Supervision, Satisfaction

## Abstract

**Objective:**

Prior to the pandemic, trainee doctors were at higher risk of psychological ill health. There is limited evidence measuring the impact of COVID-19 on psychiatry trainees. This study evaluates levels of burnout, work satisfaction, and psychological well-being in psychiatry junior doctors in Ireland and identifies potential contributing factors.

**Methods:**

The authors carried out a cross-sectional online survey measuring demographic and work-related variables. Questions including exposure to COVID-19 and stress-related factors were included. We evaluated burnout, work satisfaction, and psychological well-being using the Abbreviated-Maslach Burnout Inventory, Basic Needs Satisfaction at Work Scale, and WHO-5 Well-being Index.

**Results:**

One hundred and five doctors responded (21%). The biggest stressor reported was reduced face-to-face contact with family and friends (73%). Forty one percent reported weekly supervision changes. Sixty five percent met the criteria for burnout, compared with 36.2% in 2018. Significant factors associated with burnout included staff shortages, longer hours, and less experience. Changes in supervision and working in non-European Working Time Directive compliant rotas were associated with lower scores across all subdomains of the BNSW Scale. The WHO-5 Well-being Index identified 48% scored low in personal well-being, indicating these trainees met the threshold for depression. Changes in regular supervision (*p*=0.010) were a significant predictor of low personal well-being.

**Conclusions:**

High prevalence of burnout and low levels of well-being in this vulnerable cohort, particularly those who are inexperienced, have changes in supervision, and working longer hours is concerning. This study highlights the importance of regular supervision and support for this group.

In March 2020, healthcare systems across the globe were thrown into uncertainty when the World Health Organization declared the novel coronavirus SARS-CoV-2 a pandemic [1]. Its impact has placed considerable strain on systems that for many countries were already overwhelmed and under resourced. Healthcare workers have been forced to quickly adapt to unprecedented challenges, with little time to prepare for the potential repercussions on their psychological well-being [2].

Several studies have examined the psychological impact of working through COVID-19. These have shown that combined factors including fear of infection, worry about family, lack of access to adequate personal protective equipment (PPE), and close contact with COVID-19 cases have all been shown to increase psychological burden in healthcare workers [3]. Trainee doctors are a particularly vulnerable group to psychological ill health and burnout generally given their relative inexperience [4, 5], and their vulnerability is likely compounded by the social instability that accompanies training programs and competing demands of clinical work, research commitments, and membership examinations. There is already evidence that the ramifications of the pandemic have increased stress and burnout in this group as a whole [6, 7].

There is minimal published research available examining the impact of COVID-19 on burnout and psychological well-being specifically in psychiatry trainees. One study of psychiatry trainees during the pandemic found that 27.3% met criteria for burnout and depression, with those at a more junior stage of training being at particular risk [8]. Psychiatry trainees may be potentially more susceptible to burnout and depression in the context of exposure to trauma and suicide in daily clinical work; and it has previously been proposed that adverse psychological experiences can arise in response to the demands of training in the psychiatric environment [9]. Psychiatric services have reported a substantial increase in caseload since the onset of the pandemic [10] and it has been suggested by Hossain et al. that a “psychiatric epidemic” is occurring alongside this COVID-19 pandemic [11].

Burnout can mediate psychological well-being and is now an established occupational health syndrome characterized by three domains: mental detachment in relation to one’s job, reduced professional efficacy, and exhaustion [12]. Previous studies have shown an increased risk of burnout in psychiatry trainees associated with psychiatry not being a trainee’s first career choice, lengthy working hours, and lack of supervision, with more junior and female trainees being particularly at risk [13]. Closely related to burnout is subjective psychological well-being, an indicator for development of depressive disorder [14]. A pre-COVID-19 study of European psychiatric trainees found that the prevalence of depression stood at 20.8% [15]. A large study completed during COVID-19 found the prevalence of burnout, stress, and depression to be higher in resident physicians exposed to patients who were being tested for COVID-19 compared with residents who were not, with female residents reporting more stress [6]. While essential for all clinicians, psychological well-being arguably serves a nuanced and integral function in the work of psychiatrists, who deal with the emotional impact of major mental disorders and appraise risk to patients and the wider community, often with minimal resource allocation.

Despite its importance, there is minimal focus in the literature on the evaluation of work satisfaction in comparison to burnout and well-being. Several studies have shown that satisfaction at work strongly influences overall mental health, and impacts on burnout, self-esteem, and depression [16]. Burnout, psychological well-being, and work satisfaction have been shown to exert influence on each other in the physician setting both before and during COVID-19 [7, 17–19]. Furthermore, it has been shown that diminished work satisfaction influences burnout in psychiatry doctors specifically and is a substantial factor to attrition in training programs [19, 20].

This study aims to evaluate the impact of COVID-19 on (1) burnout, (2) work satisfaction, and (3) psychological well-being in non-consultant psychiatry doctors in Ireland, and to identify potential contributory factors. This is a follow-on study from an initial survey conducted by the authors in 2018, in which we examined similar areas of burnout, psychological well-being, and work satisfaction in a similar sample using the same validated questionnaires as this study. The initial study showed the prevalence of burnout in this sample at 36.2%, and identified that 30% of respondents scored low in personal well-being, indicating positive screening for depression. This initial study also demonstrated that lack of supervision was significantly associated with higher burnout, poorer psychological well-being, and lower work satisfaction [21]. It is hoped that the information garnered from both studies, when used along with existing evidence, will help to shape future interventions that may support the well-being of trainees, both within psychiatry services and the wider healthcare domain.

## Methods

### Participants and Setting

This was a cross-sectional, opt-in online survey as has been described previously [21]. A survey invitation was sent via email link to 510 non-consultant doctors working in psychiatry in Ireland. This invitation email contained an explanatory statement describing the survey with a link to the questionnaire. A non-consultant psychiatry doctor is any doctor working in psychiatry, either in a training or non-training post, who is not fully qualified as a consultant. In Ireland, psychiatry training is divided into two parts—Basic Specialist Training (BST) and Higher Specialist Training (HST). A non-training position is a doctor working in psychiatry who is not on a training post or qualified as a consultant, usually aiming for placement in a BST or HST post and enrolled in a Continuous Professional Development (CPD) scheme. The breakdown of the 510 invited to participate was as follows: BST *n*=270; HST *n*=124; and CPD *n*=116. Participants were registered members of the College of Psychiatrists of Ireland (CPsychI) and practiced psychiatry in Ireland. Consultants, interns, or doctors who were not practicing psychiatry were not invited to participate. Two reminder follow-up email links were sent at fortnightly intervals after the initial invite (total of three invitations). This study was approved by the Research Ethics Committee of the Royal College of Physicians of Ireland, prior to commencement. Our study was performed in accordance with appropriate data protection legislation and the Declaration of Helsinki [22]. Written informed consent was obtained from each participant prior to participating in the study.

### Questionnaire

The survey contained demographic questions on age, gender, and relationship status; and questions relating to stage of training (BST, HST, CPD scheme), years of experience in psychiatry, and if psychiatry was the preferred career choice. Participants were also asked about working hours, European Working Time Directive (EWTD) compliance, on-call hours, and supervision. The EWTD is a group of regulations designed for the health and safety of workers which are based on European Union policy and adopted by many countries in the EU. These stipulations include an average 48-h working week with appropriate rest breaks and holidays. On-call hours are defined as hours of working outside those of a regular Monday–Friday 39-h week. Questions related to COVID-19 included the following: did the respondent test positive for COVID-19 within the last 12 months; was the individual mandated to self-isolate; and level of exposure (days per week) to COVID-19-positive patients. Participants were also asked about what may have increased their stress levels during the pandemic, such as concerns regarding risk of transmission; access to adequate PPE; increased workload; staff shortages due to COVID-19; and social connectedness with family/friends. Respondents were also asked about how the pandemic had impacted supervision and leave entitlements within the last 12 months. Supervision was defined as 1 h per week face-to-face time with their appointed supervising consultant. The questionnaire also contained validated scales for assessing psychological domains of burnout, work satisfaction, and psychological well-being, using the Abbreviated-Maslach Burnout Inventory (a-MBI), the Basic Needs Satisfaction at Work (BNSW) Scale, and the WHO-5 Well-being Index. A support list was attached to the end of the survey containing links to relevant occupational well-being resources.

### Abbreviated-Maslach Burnout Inventory

The a-MBI is a reliable and validated scale for assessing burnout and correlates strongly with the longer MBI [23]. The a-MBI is a 9-item scale, with subscales examining three domains: emotional exhaustion (EE), depersonalization (DP), and personal accomplishment (PA). Each subscale is assessed by three items. For each item, there is a 7-point Likert scale which ranges from never (0) to every day (6). The score of each subscale ranges from a minimum of 0 to a maximum of 18. For emotional exhaustion and depersonalization, higher scores indicate greater levels of burnout, with the inverse applied to personal accomplishment. Validated burnout cutoffs for the a-MBI were used (>6 for depersonalization, >9 for emotional exhaustion, and <9 for personal achievement). Consistent with previous studies, respondents were defined as suffering burnout if they scored above the defined cutoff scores in either the depersonalization or emotional exhaustion categories [23].

### Basic Needs Satisfaction at Work Scale

The BNSW Scale examines three domains: “competence,” reflecting the need to feel efficient or have work tasks in hand; “autonomy,” reflecting the need to experience self-determination at work; and “relatedness,” covering the need to have meaningful relations with and feel appreciated by others [24]. These three dimensions measure intrinsic motivations driving people to satisfy basic needs at work and are a validated and reliable measure in this context [24, 25]. This scale consists of 21 items answered on a 7-point Likert scale ranging from 1 (“Not at all true”) to 7 (“Very true”). After nine items were reverse scored, the three subscale scores were calculated by averaging item responses. Scores range from 1 to 7, with a higher score indicating a greater experience of needs satisfaction at work.

### WHO-5 Well-being Index

The WHO-5 Well-being Index is a self-report measure consisting of five statements which relate to subjective psychological well-being (14). Respondents rate answers on a Likert scale ranging from 0 (“at no time”) to 5 (“all of the time”), according to how they felt over the last 2 weeks. The total raw score (0–25) is multiplied by 4 to give a final score, with higher scores indicating better well-being. The WHO-5 Well-being Index has been found to have good validity in measuring for well-being and high sensitivity and specificity in screening for depression [14, 26]. A WHO-5 cutoff score of ≤ 50 is recommended when screening for clinical depression [14].

### Analysis

All analyses were carried out on IBM Statistical Package for Social Sciences® Version 26. Demographic and work-related data were summarized using descriptive analysis. Continuous data normality was tested using the Shapiro-Wilk test. Likert scales were considered interval data and internal reliability of the scales was assessed by calculating Cronbach’s alpha coefficients for each scale. The association between categorical variables was explored using the chi-square test or Fisher’s exact test as appropriate. For continuous variables, the two-sample *t* test for parametric data and the Wilcoxon Rank-sum test for non-parametric data were used. No Bonferroni correction was applied to account for multiple testing. A *p* value <0.05 was used as the level of significance.

## Results

### Sample Profile

There were 105 (21%) respondents to the study. In terms of demographics, 63% (*n*=66) were female and 74% (*n*=78) were in a relationship. 56% (*n*=59) of respondents were on a Basic Specialist Training program; 38% (*n*=40) of respondents were on a Higher Specialist Training program; and 6% (*n*=6) were enrolled in CPD scheme. 81% (*n*=85) received regular supervision, and psychiatry was the preferred career choice for 91% (*n*=96). 90% of respondents carried out on-call duties; 63% were EWTD compliant; and 66% worked in the Emergency Department. Table 1 summarizes the baseline demographics and work-related variables.

**Table 1:** Demographic and work-related variables

			*n*	%
Age categories	*20–29*		20	19%
	*30–39*		71	68%
	*40–49*		9	9%
	*50–59*		5	5%
Gender	*Male*		39	37%
	*Female*		66	63%
Relationship status	*In a relationship*		78	74%
	*Single*		27	26%
Psychiatry preferred career	*Yes*		96	91%
	*No*		9	9%
Training level	*BST*		59	56%
	*HST*		40	38%
	*Locum*		6	6%
Experience in psychiatry	*Years*		Median 4	IQR (3–7)
Regular supervision	*Yes*		85	81%
	*No*		20	19%
On-call	*Yes*		94	90%
	*No*		11	10%
24-h on-call	*Yes*		88	84%
	*No*		17	16%
ED on-call	*Yes*		69	66%
	*No*		36	34%
EWTD compliant rota	*Yes*		66	63%
	*No*		39	37%

*BST*, Basic Specialist Training (core/junior stage of training); *HST*, Higher Specialist Training (senior stage of training); *EWTD*, European Working Time Directive. The EWTD is a group of regulations designed for the health and safety of workers—which are based on European Union policy and adopted by many countries in the EU. One of these stipulations is an average 48-h working week with appropriate rest breaks and holidays

### COVID-19-Related Factors

Fifty-five respondents (52%) had to self-isolate over the previous 12 months. Nine (.9%) individuals had tested positive for COVID-19. Fifty-three respondents (51%) reported that they were unable to take annual leave and 40 respondents (38%) could not take educational leave due to staffing shortages related to COVID-19. Forty-three respondents (41%) reported their weekly supervision changed during the pandemic. Of these, 26% reported reduced frequency of sessions, 10% reported shorter sessions, and 14% reported a change from in-person to online sessions. The remainder reported longer sessions or no change. See Table 2 for COVID-19-related factors.

**Table 2:** COVID-19-related factors

		*n*	%
Have you been working with individuals who have tested positive for COVID-19?	*Yes*	86	82%
	*No*	19	18%
Did you have to self-isolate over the past 12 months because of concerns re COVID-19 exposure?	*Yes*	55	52%
	*No*	50	48%
Did you test positive for COVID-19 over the last 12 months?	*Yes*	9	9%
	*No*	96	91%
Were you unable to take your annual leave during the last 12 months due to staffing issues related to COVID-19?	*Yes*	53	51%
	*No*	52	49%
Were you unable to take your educational leave during the last 12 months due to staffing issues related to COVID-19?	*Yes*	40	38%
	*No*	65	62%
Have your weekly supervision sessions changed during the pandemic?	*Yes*	43	41%
	*No*	62	59%

Factors that increased respondents’ stress during the pandemic are as follows: the biggest stressor was due to reduced face-to-face contact with family and friends (73%). 72% reported it was due to staff shortages and 66% reported stress increased due to higher clinical workload. 48% reported that concerns regarding risk of contracting COVID-19 increased stress and 19% reported that having inadequate access to PPE was a factor.

### Burnout a-MBI

The a-MBI was scored from 0 to 18 for each domain. Validated burnout cutoffs were used (>6 for depersonalization, >9 for emotional exhaustion, and <9 for personal accomplishment) [19]. The a-MBI identified a median score of 11 (IQR 7–14) for EE, a median score of 5 (IQR 1–8) for DP, and a median score of 13 (IQR 10.5–16) for PA among respondents.

Based on the calculated total score of emotional exhaustion and depersonalisation, burnout was present in 68 respondents (65%) of this sample. There was adequate internal validity of the subscales based on Cronbach’s alpha coefficients as presented in Table 3.

**Table 3:** a-MBI subscale scores

Subscale	Median (IQR)	Category	Frequency	Percent	Cronbach alpha
Personal achievement	13 (10.5–16)	Burnout	12	11%	0.695
		No burnout	93	89%	
Emotional exhaustion	11 (7–14)	Burnout	65	38%	0.803
		No burnout	40	62%	
Depersonalisation	5 (1–8)	Burnout	38	36%	0.779
		No burnout	67	64%	

Significant factors associated with burnout included being unable to take annual leave due to the pandemic; staff shortages due to the pandemic; and working in a rota that was non-EWTD compliant. Having less psychiatry experience was also significant, with a mean difference of 1.182 years (CI 0.178–2.186). Changes in supervision were also associated with presence of burnout, though this association only trended towards significance (*p* = 0.052).

### Basic Needs Satisfaction at Work

The three subscale scores on the Basic Needs Satisfaction at Work (BNSW) Scale were calculated by averaging item responses. Results showed that respondents had a moderate sense of autonomy (mean 3.55 ± 1.3) with a higher sense of competence (mean 4.39 ± 1.1) and relatedness (mean 4.48 ± 1.2). There was high internal validity of the subscales based on Cronbach’s alpha coefficients as presented in Table 4. Changes in supervision and working in a rota that was non-EWTD compliant were associated with lower scores across all three subdomains of the BNSW Scale. Increased workload due to COVID-19; working in the Emergency Department, and psychiatry not being the non-consultant hospital doctor’s (NCHD) preferred career choice decreased autonomy and relatedness but not competence. Lack of PPE decreased autonomy and competence but not relatedness (see Table 5).

**Table 4:** Basic Needs Satisfaction at Work Subscale scores

Subscale	Mean	SD	Cronbach’s alpha
Autonomy	3.55	1.32	0.885
Competence	4.39	1.1	0.8
Relatedness	4.48	1.17	0.889

**Table 5:** Questions significantly associated with Basic Needs Satisfaction at Work Subscale scores

Question	Answer (Yes/No)	Autonomy (mean ± SD)	Competedness (mean ± SD)	Relatedness (mean ± SD)
Increased clinical workload during the pandemic?	Yes	3.34 ± 1.32 *	4.29 ± 1.03	4.30 ± 1.14 *
	No	3.95 ± 1.26 *	4.56 ± 1.20	4.83 ± 1.14 *
Inadequate availability of personal protective equipment (PPE)?	Yes	2.89 ± 1.30 *	3.94 ± 1.00 *	4.45 ± 0.96
	No	3.70 ± 1.29 *	4.49 ± 1.10 *	4.49 ± 1.21
Emergency department cover?	Yes	3.36 ± 1.33 *	4.31 ± 1.07	4.25 ± 1.09 **
	No	3.92 ± 1.25 *	4.54 ± 1.14	4.92 ± 1.20**
Psychiatry preferred specialty?	Yes	3.65 ± 1.30 *	4.42 ± 1.12	4.57 ± 1.14 **
	No	2.49 ± 1.11 *	4.02 ± 0.79	3.47 ± 0.96 **
Have your weekly supervision sessions changed during the pandemic?	Yes	3.11 ± 1.34 **	4.13 ± 1.04 *	4.07 ± 1.18 **
	No	3.86 ± 1.23 **	4.56 ± 1.10 *	4.77 ± 1.07 **
Was your rota EWTD compliant?	Yes	3.93 ± 1.16 ***	4.57 ± 1.11 *	4.79 ± 1.00 ***
	No	2.91 ± 1.35 ***	4.07 ± 1.01 *	3.95 ± 1.25 ***

Significance: *p<0.05, **p<0.01, ***p<0.001

### Psychological Well-being WHO Index

The WHO-5 Well-being Index identified 50 respondents (48%) who scored low in personal well-being. On univariate analysis, changes in regular supervision (*p*=0.010) and not dealing with COVID-19-positive patients (*p*=0.045) were found to be significant predictors of low personal well-being. There was high internal validity of the scale as evidenced by a Cronbach alpha coefficient of 0.9.

## Discussion

The high burnout prevalence of 65% in this sample is concerning, a considerable increase from our initial study conducted prior to the COVID-19 pandemic, which showed a burnout prevalence of 36.2% [21]. Compared with current literature, a Saudi Arabian study by Alkhamees et al. [8] completed during COVID-19 showed a burnout rate of 27.3% in a sample of 121 psychiatry trainees. Reasons for such differences between the two COVID-19 studies may be related to a difference in workload or timing of COVID-19 “waves.” Hospital activity greatly reduced during the early stages of the pandemic in the study by Alkhamees et al., whereas workload and resource availability were significant (hours, staff, and lack of leave) in our study. We found that trainees with less psychiatry experience were more prone to burnout, and also found that levels of supervision play an important role, consistent with findings in this group pre-COVID-19 [13]. This result indicates that more targeted strategies for supervising inexperienced trainees may be helpful in preventing burnout.

It is important to mention that the proportion of CPD respondents was low at 6 out of 105 (5.7%). The proportion of CPD doctors invited to participate in the survey was 116 out of 510 (22%) of the total, meaning potentially valuable information about this group was not fully captured. These doctors are not on a training scheme and are group who may be even more susceptible to burnout and depression, given that they are often “on the fringes” of the system. They are also less likely to receive 1-h formal supervision per week, given that it is not a mandatory requirement unless on a training scheme. Furthermore, non-EU doctors have more difficulty in getting training places and so are more likely to be on CPD schemes—this means this group may be also more culturally or socially isolated. The doctors in this important subgroup are worth further study.

Trainees in our study reported reasonable work satisfaction. This is in keeping with satisfaction levels reported in the speciality of psychiatry as a whole [27]. It is unsurprising that non-EWTD compliance and changes in supervision decreased all three domains of the BNSW Scale. An over-stretched trainee is less likely to derive a meaningful sense of satisfaction and fulfillment from their work if they are struggling to meet basic obligations. Lack of supervision was a key association in relation to this area in our last study—a relatively unresearched finding in psychiatry trainees. The relationship between regular supervision and job satisfaction is logical—a trainee in the optimal supervision setting is more likely to feel supported in difficulty and receive constructive feedback around weaknesses and strengths, thereby improving their confidence and competence. Receiving appropriate supervision has been shown to be associated with increased job satisfaction and lower burnout in other healthcare settings outside psychiatry training [28, 29].

Regarding psychological well-being, it is striking that 48% of our sample scored low enough to meet the criteria for a positive depression screen, an increase from 30% in our pre-COVID-19 study. Our result compares with 27.3% meeting depression criteria in the COVID-10 study by Alkhamees et al. [8]. It is possible that burnout and work satisfaction may be related, and while we did not evaluate the relationship in this study, it has been shown that work dissatisfaction and personal burnout predicted low WHO-5 well-being scores in physicians [18]. It is interesting to note that not dealing with COVID-19-positive patients predicted lower well-being scores in our sample. In a similar vein, other studies during COVID-19 have found that those not on the “frontline” suffered higher burnout [30, 31]. One study of Turkish physicians found that those not “actively involved in the fight against the virus” suffered higher burnout than those who were “actively involved” [30]. While this term is very broad, it is reasonable to assume that those who did not perceive themselves to be directly engaged in COVID-19 work felt less effective than their colleagues, which may have contributed to lower sense of professional efficacy (a core burnout domain). In contrast, it has also been shown that trainee physicians exposed to COVID-19 compared with non-exposed had higher rates of burnout and stress [6]. The relationship between burnout, psychological well-being, and depressive disorder is complex, and has been described in detail by Maslach and Leiter [32]; however, it has been shown that burnout may mediate the transition to depression in the workplace [33]. Depression can lead to suicidal ideation—the Boss study reported on suicidal ideation in psychiatry trainees, which was found to be as high as 20% in some countries [13].

Supervision surfaced again as another key finding in this study in relation to predicting lower well-being, in keeping with our pre-COVID-19 study. Our study found that it was not necessarily lack of supervision, but changes to supervision (of which decreased frequency was most common) that were significant. Psychiatry as a specialty has unique challenges for trainees that necessitate close attention to regular and consistent supervision. The psychotherapeutic aspect to consultations, emphasis on rehabilitation versus “cure” for mental illness, and exposure to patient self-harm or violence may lead to trainees feeling overwhelmed in their clinical work. Qualitative research has shown that stress in psychiatry trainees is exacerbated by feeling “helpless,” “ill-equipped,” and unsupervised [9]. Ensuring there is a focus on regular good-quality supervision will likely have the cascade effect of optimizing quality patient care, as it has been shown that clinical supervision may be associated with a decrease in distressing symptoms in individuals diagnosed with a mental illness [34]. The burden on psychiatric services is increasing with COVID-19, and it is imperative that supervision is not neglected in favor of focusing resources on service provision only. Doctor migration is strongly impacted by negative training experiences such as lack of proper supervision as outlined recently by Brugha et al. [35]. It is now more essential than ever to ensure retention of trainees from the outset and avoid this negative cycle.

This study is relevant and important given the limited research to date on the impact of COVID-19 on psychiatry trainees, the importance of maintaining high training standards, and the retention difficulties for junior doctors in Ireland and elsewhere. Our study was strengthened by the use of validated scales with high internal consistency, and sheds further light on the essential and understudied role of supervision in trainee well-being, work satisfaction, and burnout. Limitations of this study include the risk of responder bias and recall bias, given that this was a self-report study. The small sample size and somewhat low response rate could also be regarded as limitations. We did not have data on other factors that could further influence our findings, such as mental health history.

Supervision in psychiatry is an area worthy of more research [36]. The trainer/trainee relationship in supervision is one of the most important aspects to supervision in clinical systems [37]. Trainers themselves need to be supported systemically to ensure they have adequate resources to provide quality training and supervision, as addressed by the CAP-STATE study, which evaluates the diversity across training institutions in Europe and recognizes the need for appropriate governance and support of such institutions [38]. Meeting basic needs such as adequately staffing teams and adherence to leave entitlements is important in ensuring psychological well-being; and cognitive behavioral interventions and Schwartz rounds have been shown to impact positively on these areas if implemented properly [39].

In conclusion, our study shows that during the COVID-19 pandemic psychiatry trainees are experiencing higher rates of burnout and lower rates of psychological well-being, and those who are inexperienced have changes in supervision, and working longer hours without sufficient resources are suffering more. The psychological impact of COVID-19 affects every area of the healthcare sector, and trainees are a particularly vulnerable group. Their supervision and support are essential, not only so they feel valued and connected, but so they can progress in training and remain within the workforce. In the context of predicted increased pressures on psychiatry services as the pandemic continues, continuing to deliver high-quality care while protecting those that deliver it has never been more crucial.
